# *Sac*Pox from the thermoacidophilic crenarchaeon *Sulfolobus acidocaldarius* is a proficient lactonase

**DOI:** 10.1186/1756-0500-7-333

**Published:** 2014-06-03

**Authors:** Janek Bzdrenga, Julien Hiblot, Guillaume Gotthard, Charlotte Champion, Mikael Elias, Eric Chabriere

**Affiliations:** 1 URMITE UMR CNRS-IRD 6236, IFR48, Faculté de Médecine et de Pharmacie, Université de la Méditerranée, Marseille, France; 2 Weizmann Institute of Science, Biological Chemistry, Rehovot, Israel

**Keywords:** Lactonase, PLL, *Quorum* sensing, Phosphotriesterase, Extremophile, Thermoacidophile

## Abstract

**Background:**

*Sac*Pox, an enzyme from the extremophilic crenarchaeal *Sulfolobus acidocaldarius* (*Sac*), was isolated by virtue of its phosphotriesterase (or paraoxonase; Pox) activity, *i.e.* its ability to hydrolyze the neurotoxic organophosphorus insecticides. Later on, *Sac*Pox was shown to belong to the Phosphotriesterase-Like Lactonase family that comprises natural lactonases, possibly involved in *quorum* sensing, and endowed with promiscuous, phosphotriesterase activity.

**Results:**

Here, we present a comprehensive and broad enzymatic characterization of the natural lactonase and promiscuous organophosphorus hydrolase activities of *Sac*Pox, as well as a structural analysis using a model.

**Conclusion:**

Kinetic experiments show that *Sac*Pox is a proficient lactonase, including at room temperature. Moreover, we discuss the observed differences in substrate specificity between *Sac*Pox and its closest homologues *Sso*Pox and *Sis*Lac together with the possible structural causes for these observations.

## Background

**Figure 1:**
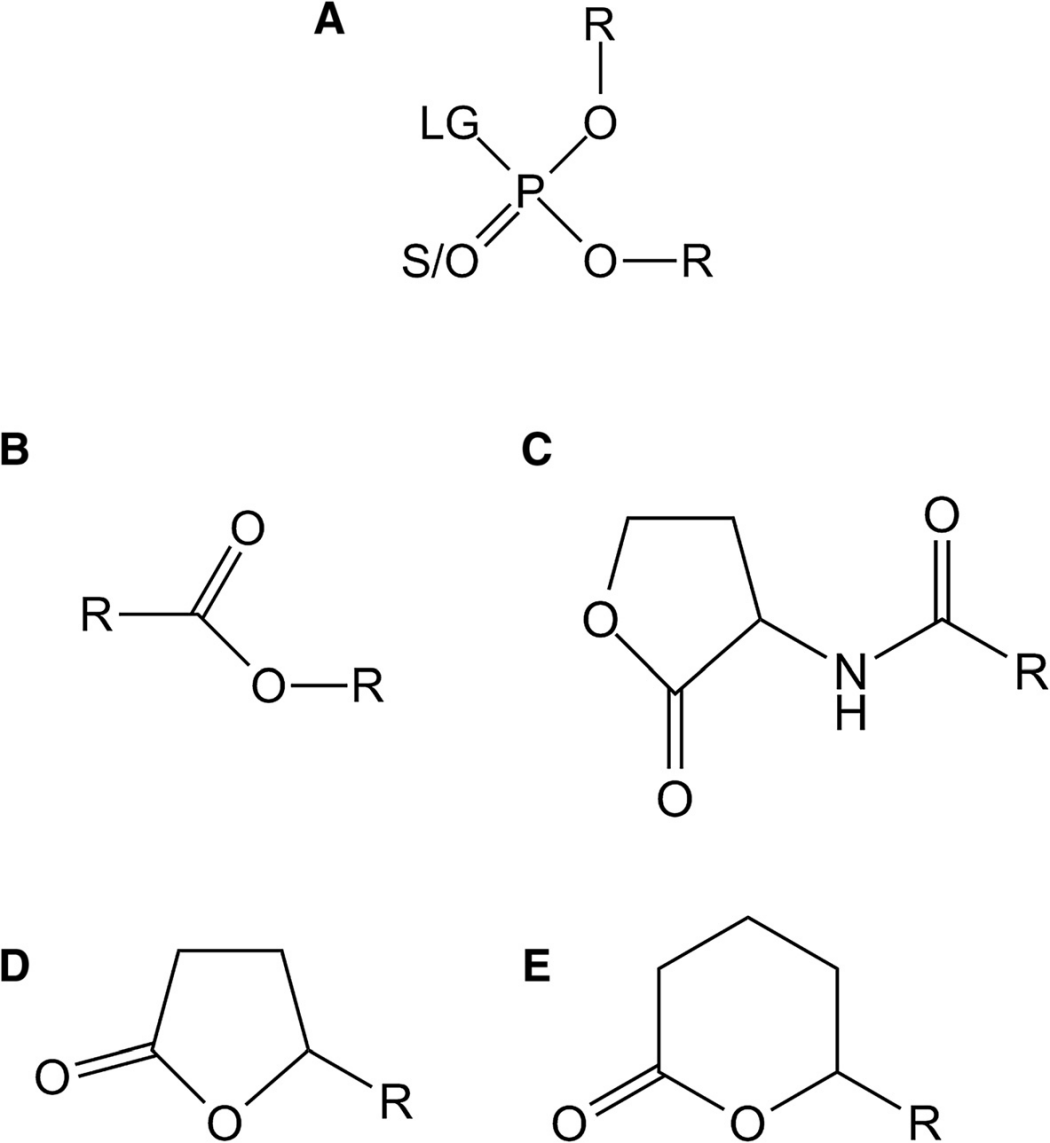
**Chemical structure of *Sac*Pox substrates.** Chemical structures of **(A)** phosphotriesters, **(B)** esters, **(C)** Acyl-Homoserine Lactones, **(D)** γ-lactones and **(E)** δ-lactones are presented. For phosphotriesters, R corresponds to different nature of substituents; LG corresponds to the leaving group. The terminal substituent could be S atom if the molecule is a thionophosphotriester or an O atom if the molecule is an oxonophosphotriester. For esters, R corresponds to different nature of substituent. For AHLs and γ/δ-lactones, R corresponds to different size of acyl chain.

Phosphotriesterase-Like Lactonases (PLLs) are natural lactonases (EC 3.1.1.25) (Figure 1C, D, E) with promiscuous phosphotriesterase activity (EC 3.1.8.1) (Figure 1A) [1,2]. They are structurally closely related to bacterial phosphotriesterases (PTEs) [3-6], such as *Brevundimonas diminuta* PTE (*Bd*PTE; ~30% sequence identity) [7]. PTEs naturally hydrolyze neurotoxic organophosphorus (OPs) compounds (Figure 1A) such as paraoxon (the active metabolite of the insecticide parathion) with catalytic constants that approach the diffusion limit (*i.e.* k_cat_/K_M_ ~ 10^8^ M^−1^ s^−1^) [7]. Because OPs have been massively used as pesticides since the 50′s [8], PTEs are believed to have emerged in few decades from a PLL progenitor [2], providing a new source of phosphorus to bacteria, and consequently a selective advantage [8].

Both enzyme families exhibit the same (β/α)_8_-barrel topology [9,10] and belong to the amidohydrolase superfamily [11,12]. Their structure consists of 8 β-strands forming a central barrel surrounded by 8 α-helixes. The active site is constituted by a bimetallic center (two metal cations) localized at the C-terminus of the barrel. Metal cations are coordinated by four histidines, an aspartic acid and a carboxylated lysine residue [9]. While the nature of the bimetallic center can vary depending on the enzyme nature and the purification procedure [3,5,13,14], the catalytic mechanism is presumed to be identical. The bimetallic center activates a water molecule into a hydroxide ion which performs a nucleophilic attack onto the electrophilic center [9,15].

The difference in substrate specificities of PLLs and PTEs seems mainly governed by variation in the connecting loops of the barrel [2,16]. Major differences between PTEs and PLLs reside in the active site loop size and conformation [1,2]. Indeed, loop 7 is shorter in PLLs than in PTEs whereas the loop 8 is larger, forming a hydrophobic channel that accommodates lactones aliphatic chain [9]. Loop 7/8 length and sequence also differ within the PLL family and led to the identification of two different subfamilies: PLLs-A and PLLs-B [2]. Both subfamilies exhibit different substrate specificities: PLLs-B are exclusively oxo-lactonases (Figure 1DE) whereas PLLs-A hydrolyze efficiently oxo-lactones and Acyl-Homoserine Lactones (AHLs, Figure 1C) [2]. AHLs are messenger molecules involved in a bacterial communication system dubbed *quorum* sensing (QS) [17]. QS regulates the expression of numerous genes, and enables bacterial population to adopt a “group” behavior, including the expression of virulence factors of some pathogens [18,19]. The involvement of PLLs-A in *quorum* sensing has not yet been demonstrated, and these enzymes are often found with no other AHL components, including in archaeal species [20]. However, the fact that they hydrolyze specifically the natural enantiomer of AHL indicates that it may be their native substrate [16].

PLLs are promiscuous enzymes that catalyze two chemical reactions of potential biotechnological interest. Indeed, the inhibition or “quenching” of the QS is seen as a possibly promising strategy to develop innovative therapies [21-25]. Indeed, lactonases such as PLLs can inhibit QS (known as *quorum* quenching, *i.e.* QQ) [26,27] and thereby annihilate the virulence of micro-organisms possessing an AHL-based QS system [28]. Moreover, PLLs are endowed with relatively low phosphotriesterase activity, but might be optimized against OPs and subsequently used for degrading organophosphorus pesticides [3,5,6,9,29] and nerve agents [30], for which no satisfactory remediation methods are currently available [31].

In addition, several PLLs members are thermostable [3,4,6,32-34]; *e.g.* PLLs from extremophilic crenarchaeaon sources [3,4,16,34]. These counterparts exhibit industry-compatible properties (*e.g.* thermal and detergent resistance) [35-37]; making them good starting point for *in vitro* improvement protocols [37,38]. Several studies report the engineering of thermostable PLLs and improvement of catalytic efficiency against OPs, including for *Sso*Pox [16,39], *Dr*OPH (*Deinococcus radiodurans* organophosphorus hydrolase) [6,40] and *Gk*L (*Geobacillus kaustropilus* lactonase) [41] but also for the lactonase activity of *Sso*Pox [16], MCP (*Mycobacterium avium* subsp. Paratuberculosis K-10 lactonase) [42] and *Gk*L [43].

Here we focus on *Sac*Pox, the PLL from the thermoacidophilic crenarchaeon *Sulfolobus acidocaldarius* (living conditions: 55–85°C, pH 2–3) [44]. *Sac*Pox was originally isolated and studied for its ability to hydrolyze OP compounds at high temperature [4]. The enzyme shares about 30% of sequence identity with *Bd*PTE and about 70% with its closest homologues, *i.e. Sso*Pox from *Sulfolobus solfataricus*[3] and *Sis*Lac from *Sulfolobus islandicus*[33,45]. Being an enzyme from a hyperthermophile, *Sac*Pox is however less stable than *Sso*Pox (half-life of 5 min at 90°C [4] and of 4 h at 95°C [3,46], respectively). The kinetic characterizations performed on *Sac*Pox revealed that it hydrolyzes OP, ester and lactone molecules at high temperature [4,13]. However, only few substrates have been tested, and no natural lactones were assayed as substrate. In this study, we performed a broad kinetic characterization of *Sac*Pox at room temperature (25°C) for several OPs, esters (Figure 1B) and lactone molecules including AHLs, γ-lactones and δ-lactones in the aim to evaluate the biotechnological potentialities of this enzyme.

## Methods

### Sequence alignment

The sequence alignment was performed based on the previously published PLL sequence alignment [2], using the *T-coffee* server (expresso) [47,48] and manually improved with the *seaview* software [49]. It contains 29 different sequences (Additional file 1: Table S1). The sequence alignment was represented using the *BioEdit* 7.1.3 software [50]. Protein sequence identities were computed using *ClustalW* server [51]. The phylogenetic tree was performed using *PhyML*[49] and default parameters.

### Protein production and purification

The protein production and subsequent purification steps were performed analogously to previously described [16,33,34,45,52-54]. In brief, the protein was heterologously produced in *Escherichia coli* strain BL21(DE_3_)-pGro7/GroEL (TaKaRa) at 37°C in ZYP medium [55]. When OD_600nm_ reaches 0.8, protein production was induced with addition of arabinose (0.2%, w/v) and CoCl_2_ (2 mM) and temperature transition to 25°C for 20 hours. Cells were harvested by centrifugation, and pelleted cells were suspended in *lysis buffer* (50 mM HEPES pH 8, 150 mM NaCl, 0.2 mM CoCl_2_, lysozyme 25 mg/ml, PMSF 0.1 mM, DNase I 10 mg/ml), stored at −80°C during 2 hours; then sonicated 3 times during 30 seconds (Branson Sonifier 450, 80% intensity and microtype limit of 8) and centrifuged. Taking advantage of the high stability of *Sac*Pox, the supernatant was heated at 70°C during 30 minutes and centrifuged before proceeding a STREP-TRAP affinity chromatography step (GE Healthcare, Uppsala, Sweden). The sample was then cleaved by the Tobacco Etch Virus protease (TEV, ratio 1:20, w/w [56]) during 20 hours at 30°C prior to be loaded a second time on STREP-TRAP affinity chromatography. The flow through containing the cleaved protein was then concentrated and loaded on a size exclusion column (S75-16-60; GE Healthcare, Uppsala, Sweden). The protein purity and identity were checked by SDS-PAGE and mass spectrometry analysis (MS platform Timone, Marseille, France). The protein concentration was determined using a nanospectrophotometer (Nanodrop, Thermofisher Scientific, France) using its molar extinction coefficient (*Sac*Pox ϵ_280 nm_ = 35 307.7 M^−1^ cm^−1^) calculated by the *PROT-PARAM* server [57].

### Kinetic characterization

#### General procedures

Catalytic parameters were evaluated at 25°C and recorded with a microplate reader (Synergy HT, BioTek, USA) and the Gen5.1 software as previously explained [16,33,52,54]. The reaction was performed in a 200 μL volume using a 96-well plate with a 6.2 mm path length as previously described [33]. The collected data were subsequently fitted to the Michaelis-Menten (MM) equation [58] using *Graph-Pad Prism 5.00* (GraphPad Software, San Diego California USA, http://www.graphpad.com). In cases where V_max_ could not be reached, the catalytic efficiency was obtained by fitting the linear part of MM plot to a linear regression using *Graph-Pad Prism 5.00* software.

#### OP hydrolase and esterase kinetics

Standard assays for organophosphates (Figure 1A) and esters (Figure 1B) were performed in *activity buffer* (50 mM HEPES pH 8, 150 mM NaCl, 0.2 mM CoCl_2_) by measuring the *p*-nitrophenolate release over time at 405 nm (ϵ_405 nm_ = 17 000 M^−1^ cm^−1^). For ethyl-paraoxon (Additional file 1: Figure S1I), the *activity buffer* has also been supplemented with SDS (w/v) at 0.01% or 0.1% for detergent essays. Malathion (Additional file 1: Figure S1V) hydrolysis was followed at 412 nm in *activity buffer* added of 2 mM DTNB to follow the release of free thiols (ϵ_412 nm_ = 13 700 M^−1^ cm^−1^). The time course hydrolysis of dihydrocoumarin (Additional file 1: Figure S1X), CMP-coumarin (Additional file 1: Figure S1VI) and phenyl-acetate (Additional file 1: Figure S1VII) were respectively monitored at 270 nm (ϵ_270 nm_ = 1 400 M^−1^ cm^−1^), 412 nm (ϵ_412 nm_ = 37 000 M^−1^ cm^−1^) and 270 nm (ϵ_270 nm_ = 1 400 M^−1^ cm^−1^).

#### Lactonase kinetics

Kinetics monitoring the lactone hydrolysis were performed according to a previously described protocol [33]. The lactone hydrolysis was monitored in the *lactonase buffer* (2.5 mM Bicine pH 8.3, 150 mM NaCl, 0.2 mM CoCl_2_, 0.25 mM Cresol purple and 0.5% DMSO) with different AHLs (Figure 1C) [*i.e.* C4-AHL (*r*), C6-AHL (*r*), C8-AHL (*r*), 3-oxo-C8-AHL (*l*), 3-oxo-C10-AHL (*l*)] (Additional file 1: Figure S1XI-XVI) and oxo-lactones (Figure 1D,E) [*i.e.* ϵ-caprolactone, γ-heptanolide (*r*), Nonanoic-γ-lactone (*r*), Nonanoic-δ-lactone (*r*), Undecanoic-γ-lactone (*r*), Undecanoic-δ-lactone (*r*), Dodecanoic-γ-lactone (*r*) and Dodecanoic-δ-lactone (*r*)] (Additional file 1: Figure S1XVII-XXIV). Cresol purple (pK_a_ 8.3 at 25°C) is a pH indicator (577 nm) used to monitor the acidification of the medium following lactone ring hydrolysis (ϵ_577nm_ = 5 500 M^−1^ cm^−1^).

### Structural modeling and structural analysis

The *Sac*Pox structure was modelled using the *ESyPred3D* server using *Sac*Pox protein sequence as query and *Sso*Pox structure (2VC5) as template [59]. Structures were analyzed and figure made using PyMol [60].

## Results

**Figure 2:**
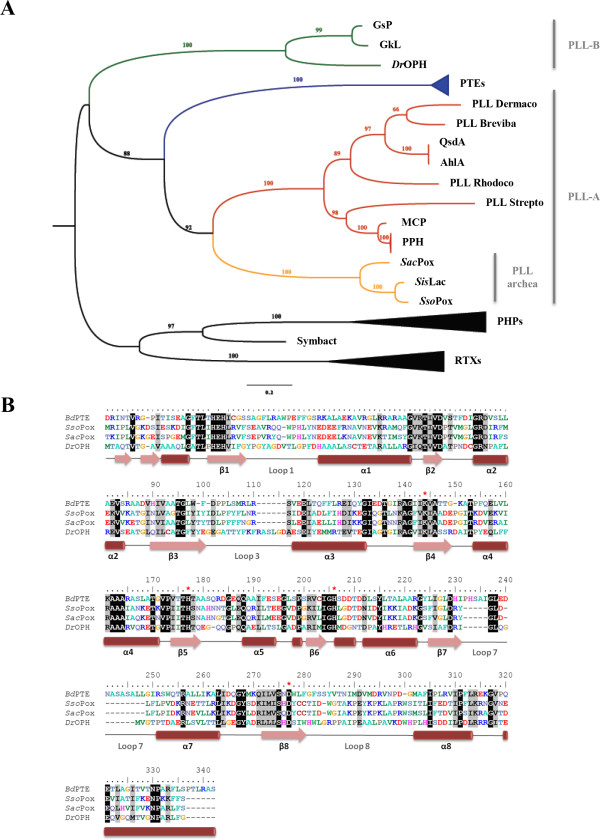
**Phylogenetic analysis of the PLL family. A**. Phylogenetic tree of PLLs, PTEs, and close homologues. Members of PLL-B are colored in green while within the PLL-As, mesophilic and archaeal PLLs are respectively colored in red and orange. The clades of PHPs, PTEs and RTXs were collapsed for clarity. All the sequences used for this tree are listed in Additional file 1: Table S1. **B**. Sequence alignment of *Bd*PTE from *B. diminuta*, *Sso*Pox from *S. solfataricus*, *Sac*Pox from *S. acidocaldarius* and *Dr*OPH from *D. radiodurans*. Conserved amino acid residues are highlighted in black and similar residues in grey. Conserved active site residues involved in metals coordination are highlighted by red stars. Secondary structures are represented according to *Sso*Pox structure (with pink arrows depicting β-sheets and red cylinders depicting α-helixes).

First classified within the bacterial PTEs, *Sac*Pox shares in fact only 33.8% sequence identity with *Bd*PTE (Additional file 1: Table S2). *Sac*Pox indeed belongs to the PLLs-A (Figure 2A) [2]: it shares 76.1% of sequence identity with its closest homologues *Sso*Pox and *Sis*Lac, and only 30.6% identity with the PLL-B *Dr*OPH. Together with *Sis*Lac and *Sso*Pox, *Sac*Pox comprises the creanarcheal clade of the PLLs-A (Figure 2A). The sequence alignment highlights the strict conservation of essential active site residues between the different clades (Figure 2B).

### Enzymatic characterization

#### Phosphotriesterase activity

*Sac*Pox ability to hydrolyze insecticides ethyl/methyl-paraoxon, ethyl/methyl-parathion and malathion has been evaluated (Table 1). The best *Sac*Pox phosphotriester substrate, methyl-paraoxon is processed with moderate catalytic efficiency (k_cat_/K_M_ = 1.10(±0.17)×10^3^ M^−1^.s^−1^), low rate (k_cat_ = 0.307 s^−1^) and low K_M_ (278.3 μM). Very similar catalytic efficiencies were recorded for *Sso*Pox and *Sis*Lac: k_cat_/K_M_ of 1.27×10^3^ M^−1^.s^−1^ and 4.26×10^3^ M^−1^.s^−1^, respectively [33,52]. Ethyl-paraoxon comprise a slower substrate, (k_cat_/K_M_ = 2.81×10^2^ M^−1^.s^−1^), highlighting the enzyme preference for OP substrates with small substituents. No hydrolysis could be measured for ethyl-parathion and malathion, whereas a low catalytic efficiency was recorded for methyl-parathion (k_cat_/K_M_ = 4.31 M^−1^.s^−1^). This specificity profile illustrates the clear preference of *Sac*Pox for oxono-phosphotriesters rather than thiono-phosphotriesters; as previously observed for *Sso*Pox [52] and *Sis*Lac [33]. Moreover, whereas anionic detergents like SDS can significantly stimulate *Sso*Pox phosphotriesterase activity [52], the same treatment on *Sac*Pox yields only a 2-fold increase in catalytic efficiency with ethyl-paraoxon as substrate. Finally, we show that *Sac*Pox hydrolyzes CMP-coumarin (k_cat_/K_M_ = 4.38 × 10^2^ M^−1^.s^−1^), albeit with 20-fold lower catalytic efficiency than *Sso*Pox [52].

**Table 1:** Phosphotriesterase kinetic parameters

	**k**_**cat **_**(s**^**−1**^**)**	**K**_**M **_**(μM)**	**k**_**cat**_**/K**_**M **_**(M**^**−1**^**.s**^**−1**^**)**
**Paraoxon**	0.12 ± 0.01	434 ± 54	2.81 (±0.38) × 10^2^
**Paraoxon 0.01% SDS**	0.28 ± 0.01	537 ± 48	5.22 (±0.51) × 10^2^
**Paraoxon 0.1% SDS**	0.25 ± 0.01	405 ± 21	6.10 (±0.34) × 10^2^
**Methyl Paraoxon**	0.31 ± 0.02	278 ± 40	1.10 (±0.17) × 10^3^
**Parathion**	ND	ND	ND
**Methyl Parathion**	ND	ND	4.31 ± 0.20
**Malathion**	ND	ND	ND
**CMP-Coumarin**	0.28 ± 0.02	642 ± 89	4.38 (±0.68) × 10^2^

ND correspond to Not Detected hydrolysis. Results have been obtained with cobalt as cofactor.

#### Esterase activity

The ability of *Sac*Pox to hydrolyze phenyl-acetate, *p*NP-acetate and *p*NP-decanoate (Additional file 1: Figure S1VII-IX) has been evaluated (Table 2). While no activity could be detected against *p*NP-decanoate, *Sac*Pox exhibits low catalytic efficiencies against both phenyl-acetate and *p*NP-acetate (k_cat_/K_M_ ≈ 50 M^−1^.s^−1^). This weak activity against classical esters differs from previous studies on the close homologues *Sso*Pox and *Sis*Lac, for which activity has only been recorded on *p*NP-acetate [33].

**Table 2:** Esterase kinetic parameters

	**k**_**cat **_**(s**^**−1**^**)**	**K**_**M **_**(μM)**	**k**_**cat**_**/K**_**M **_**(M**^**−1**^**.s**^**−1**^**)**
**Phenyl-acetate**	0.35 ± 0.05	8 181 ± 1750	42.3 ± 11.1
***p*** **NP-acetate**	0.13 ± 0.01	2 107 ± 313	60.1 ± 9.9
***p*** **NP-decanoate**	ND	ND	ND

ND correspond to Not Detected hydrolysis. Results have been obtained with cobalt as cofactor.

#### Lactonase activity

The catalytic parameters of *Sac*Pox for various lactone substrates have been measured, including against oxo-lactones (lipophilic aroma), AHLs and dihydrocoumarin (Table 3). Our results indicate a preference of *Sac*Pox for oxo-lactone substrates; *i.e.* γ-heptanolide and nonanoic-γ-lactone (k_cat_/K_M_ ≈ 2.5×10^4^ M^−1^.s^−1^), while AHLs are about 10 times worse substrates (*i.e.*; C8 AHLs, k_cat_/K_M_ ≈ 5×10^3^ M^−1^.s^−1^). Furthermore, it seems that *Sac*Pox prefers AHLs *vs* 3-oxo-AHLs since the K_M_ for C8 aliphatic chains is 5-fold lower than that for 3-oxo-C8 AHLs. Overall, long aliphatic chain substrates AHLs are better substrates for the enzyme. Indeed, short aliphatic chain AHLs are not hydrolyzed by *Sac*Pox. Interestingly, this preference is not retained for oxo-lactones, for which molecules with short or without aliphatic chain are efficiently hydrolyzed (k_cat_/K_M_ ≈ 10^4^ M^−1^.s^−1^). As previously observed for *Sso*Pox and *Sis*Lac [16,33], this feature may reveal a potential alternative binding mode of these compounds in *Sac*Pox active site. Finally, contrary to *Sso*Pox and *Sis*Lac [16,33], *Sac*Pox does not hydrolyze dihydrocoumarin.

**Table 3:** Lactonase kinetic parameters

	**k**_**cat **_**(s**^**−1**^**)**	**K**_**M **_**(μM)**	**k**_**cat**_**/K**_**M **_**(M**^**−1**^**.s**^**−1**^**)**
**C4 AHL**	ND	ND	ND
**C6 AHL**	ND	ND	ND
**C8 AHL**	0.94 ± 0.02	178 ± 26	5.28 (±0.77) × 10^3^
**3-oxo C6 AHL**	ND	ND	ND
**3-oxo C8 AHL**	0.89 ± 0.07	836 ± 178	1.07 (±0.25) × 10^3^
**3-oxo C10 AHL**	1.03 ± 0.04	213 ± 33	4.88 (±0.77) × 10^3^
**γ heptanolide**	10.25 ± 0.50	388 ± 62	2.64 (±0.44) × 10^4^
**Nonanoic-γ-lactone**	2.64 ± 0.07	109 ± 19	2.44 (±0.44) × 10^4^
**Undecanoic-γ-lactone**	0.34 ± 0.01	578 ± 78	5.89 (±0.84) × 10^2^
**dodecanoic-γ-lactone**	0.53 ± 0.03	242 ± 60	2.21 (±0.57) × 10^3^
**Nonanoic-δ-lactone**	4.55 ± 0.21	348 ± 53	1.31 (±0.21) × 10^4^
**Undecanoic-δ-lactone**	1.05 ± 0.05	168 ± 37	6.22 (±1.40) × 10^3^
**Dodecanoic-δ-lactone**	3.34 ± 0.07	185 ± 27	1.81 (±0.27) × 10^4^
**ϵ caprolactone**	15.04 ± 0.47	1 031 ± 83	1.46 (±0.13) × 10^4^
**Dihydrocoumarine**	ND	ND	ND

ND correspond to Not Detected hydrolysis. Results have been obtained with cobalt as cofactor.

### Structural analysis

Numerous attempts to crystallize *Sac*Pox were made, with no success (Elias, Hiblot, Gotthard & Chabriere, unpublished). A previous structural model was generated by homology modeling based on *Bd*PTE structure [4] (~33.8% sequence identity with *Sac*Pox), but yielded little insights given the moderate sequence identity with the template and the very significant differences in the active site loops between these two representatives of distinct enzyme families [1,9,16]. Here we generated a homology-based model using the structure of *Sso*Pox as template (76.1% of sequence identity; Additional file 1: Table S2).

**Figure 3:**
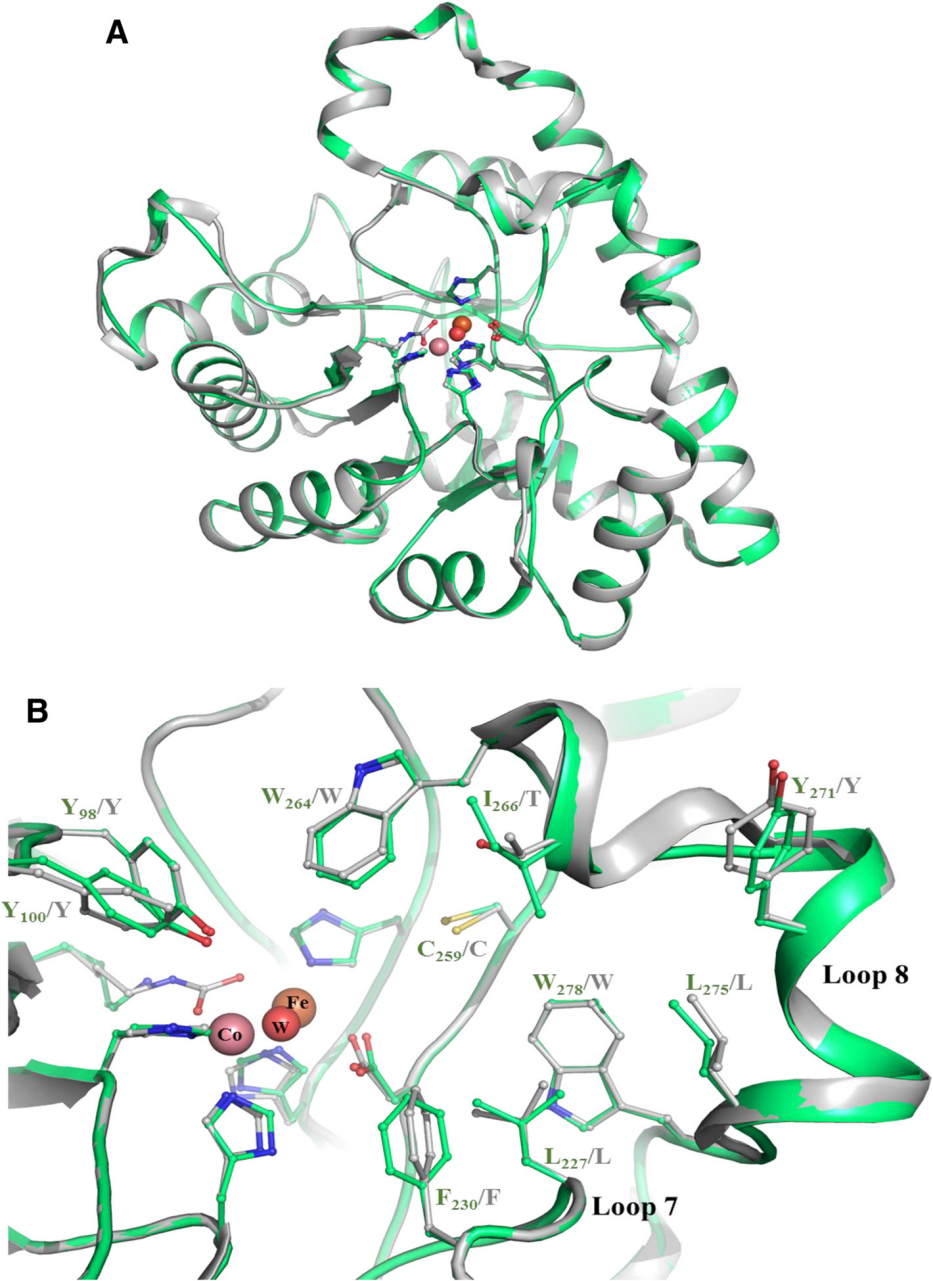
**Structural model of *Sac*Pox. A**. Structural superposition of *Sso*Pox structure (2VC5; grey) and the *Sac*Pox model (green). Cobalt, iron and the catalytic water molecule are respectively represented by pink, orange and red spheres. Bimetallic center coordinating residues are represented as sticks. **B**. Active site view of superimposed *Sso*Pox structure (grey) and the *Sac*Pox model (green). Several active site residues are represented as sticks. Numbering is made according to *Sac*Pox sequence.

As expected, the *Sac*Pox model structure almost perfectly superimposes to the *Sso*Pox crystal structure (Figure 3A). Residues forming the active site are all conserved and residues involved in loops 7 and 8 occupy nearly identical conformation in *Sac*Pox and *Sso*Pox but also in *Sis*Lac structures (Figure 3B). Noteworthy, loop 8 is partially structured into an α-helix, as seen in X-ray structures of *Sso*Pox and *Sis*Lac. A substitution (I266 in *Sac*Pox; T265 in *Sso*Pox and *Sis*Lac) in loop 8 may slightly alter the shape of the aliphatic channel. But overall, the active site of *Sac*Pox and *Sso*Pox are nearly identical (Figure 2B). Furthermore, four other substitutions between *Sac*Pox and its close homologues can be seen in loop 8: *Sac*Pox exhibits a K at position 268, instead of an R residue (R267 in *Sis*Lac), Y271 instead of L (L270 in *Sis*Lac), K278 instead of R (R277 in both *Sis*Lac and *Sso*Pox), and M281 instead of I (I280 in *Sso*Pox) (Additional file 1: Figure S2). While the structural model suggests that these substitutions are not affecting directly the binding cleft of *Sac*Pox, they might modulate loop 8 conformation and its dynamics. Indeed, it was shown in the close homologue *Sso*Pox that a single substitution in loop 8 (W263 in *Sso*Pox, equivalent to W264 in *Sac*Pox) increases the conformational flexibility of loop 8, thereby conferring higher promiscuity to the enzyme [16]. The effect is in fact so dramatic that the substitution in *Sso*Pox of W263 by any of the 19 other natural amino acids yields a variant with improved phosphotriesterase activity [16]. Additionally, loop 8 being involved in the accommodation of the aliphatic substituent of lactones substrates [9], mutations in this loop can also affect the lactonase activity [16].

## Discussion

Here we show that *Sac*Pox is a proficient lactonase (~10^4^ M^−1^.s^−1^) and can hydrolyze both oxo-lactones and AHLs. Nevertheless, *Sac*Pox have a slightly different substrate specificity than its close homologues [16,33]. Indeed, *Sac*Pox exhibits slightly lower catalytic efficiencies, prefers AHLs over 3-oxo-AHLs and does not show any activity against dihydrocoumarin. Interestingly, as noted for *Sis*Lac and *Sso*Pox [16,33], *Sac*Pox clearly prefers long chain AHLs, but can efficiently hydrolyze short chain or oxo-lactones without aliphatic substituents. This feature could reflect a putatively different binding mode of AHLs and oxo-lactones into PLLs active sites. We note that the biological role of lactonases such as PLLs is yet unclear, especially in extremophilic archaea where no AHL-based *quorum* sensing systems have been identified so far.

*Sac*Pox also exhibits promiscuous esterase and phosphotriesterase activities, a common feature of PLLs. Similarly to *Sso*Pox and *Sis*Lac [33,52], *Sac*Pox prefers OPs with small substituents. Moreover, *Sac*Pox also shows a clear preference for oxono-phosphotriesters, rather than thiono-phosphotriesters, a feature previously dubbed thiono-effect [52]. Interestingly, *Sso*Pox, *Sis*Lac and *Sac*Pox exhibit similar catalytic efficiencies against OPs (10^2–3^ M^−1^.s^−1^) at 25°C, efficiencies that are close to those measured at much higher temperatures [4].

The structural model shows that *Sac*Pox structure is very close to that of *Sso*Pox (Figure 2A). Most critically, the active sites of both enzymes are essentially identical (Figure 2B), with the exception of position 266 (I in *Sac*Pox, T in *Sso*Pox and *Sis*Lac). This substitution might partly account for the observed differences in substrates specificity between these enzymes, and would thereby represent an interesting target for future mutagenesis studies. But four other substitutions in loop 8 between these close homologues might be involved as well, and comprise also interesting options for mutagenesis studies (K268R, Y27IL, K278R and M281I). A recent study on *Sso*Pox highlighted how profound the effect on catalysis of a single substitution on loop 8 (W263) can be [16]. Therefore, substitution T266I, and/or the four others on loop 8, might contribute to the observed differences between *Sac*Pox and *Sso*Pox in substrate specificity, in combination with other factors that cannot be assessed by a structural model such as subtle changes in active site loops conformation and dynamics [16,33]. Indeed, the observed differences in the detergent stimulation between both enzymes (*Sac*Pox is only weakly stimulated by SDS, as compared to *Sso*Pox) could well be a manifestation of different dynamics of their respective active site loops.

## Conclusions

To conclude, we here demonstrate that albeit being initially isolated, characterized, and named after its ability to degrade the insecticide paraoxon (pox; [4]), *Sac*Pox is putatively a native lactonase, capable of hydrolyzing these compounds with significant catalytic efficiencies at 25°C (up to 10^4^ M^−1^.s^−1^). The extensive kinetic characterization reveals some substrate specificity differences between *Sac*Pox and its close homologues *Sis*Lac and *Sso*Pox, and the proposed structural model of *Sac*Pox suggests putative candidates (*e.g.* I266) that could account for these observations. Such positions might constitute interesting targets for future engineering studies, with the aim of improving or altering the catalytic properties of *Sac*Pox.

## Competing interests

The authors declare that they have no competing interests.

## Authors’ contributions

JH, GG and ME planed the experiments. JB, CC performed the experiments. JH, JB, ME and EC analysed the results. JB, JH and ME wrote the paper. All the authors offered a critical review of the paper.

## Supplementary Material

Additional file 1: Figure S1 Chemical structure of phosphoesters (I-VI), esters (VII-IX) and lactones (X-XXIV). **Figure S2.** Superposition of *Sso*Pox, *Sis*Lac and *Sac*Pox structural models. **Table S1.** Accession numbers of the sequences used in the phylogeny study. **Table S2.** Sequence identity matrix. Click here for file
