# Supplementary material for: SacPox from the thermoacidophilic crenarchaeon Sulfolobus acidocaldarius is a proficient lactonase
Source: BMC Res Notes. 2014 Jun 3;7:333. doi: 10.1186/1756-0500-7-333 (PMC4068969; doi:10.1186/1756-0500-7-333)
Supplement: Additional file 1: Figure S1 — Chemical structure of phosphoesters (I-VI), esters (VII-IX) and lactones (X-XXIV). Figure S2. Superposition of SsoPox, SisLac and SacPox structural models. Table S1. Accession numbers of the sequences used in the phylogeny study. Table S2. Sequence identity matrix. [file 1756-0500-7-333-S1.docx]

SUPPLEMENTAL MATERIAL FOR

***Sac*Pox from the thermoacidophilic crenarchaeon *Sulfolobus acidocaldarius* is a proficient lactonase**

**Janek BZDRENGA^1✝^, Julien HIBLOT^1✝^, Guillaume GOTTHARD^1^, Charlotte CHAMPION^1^, Mikael ELIAS^2*^ & Eric CHABRIERE^1*^**

**
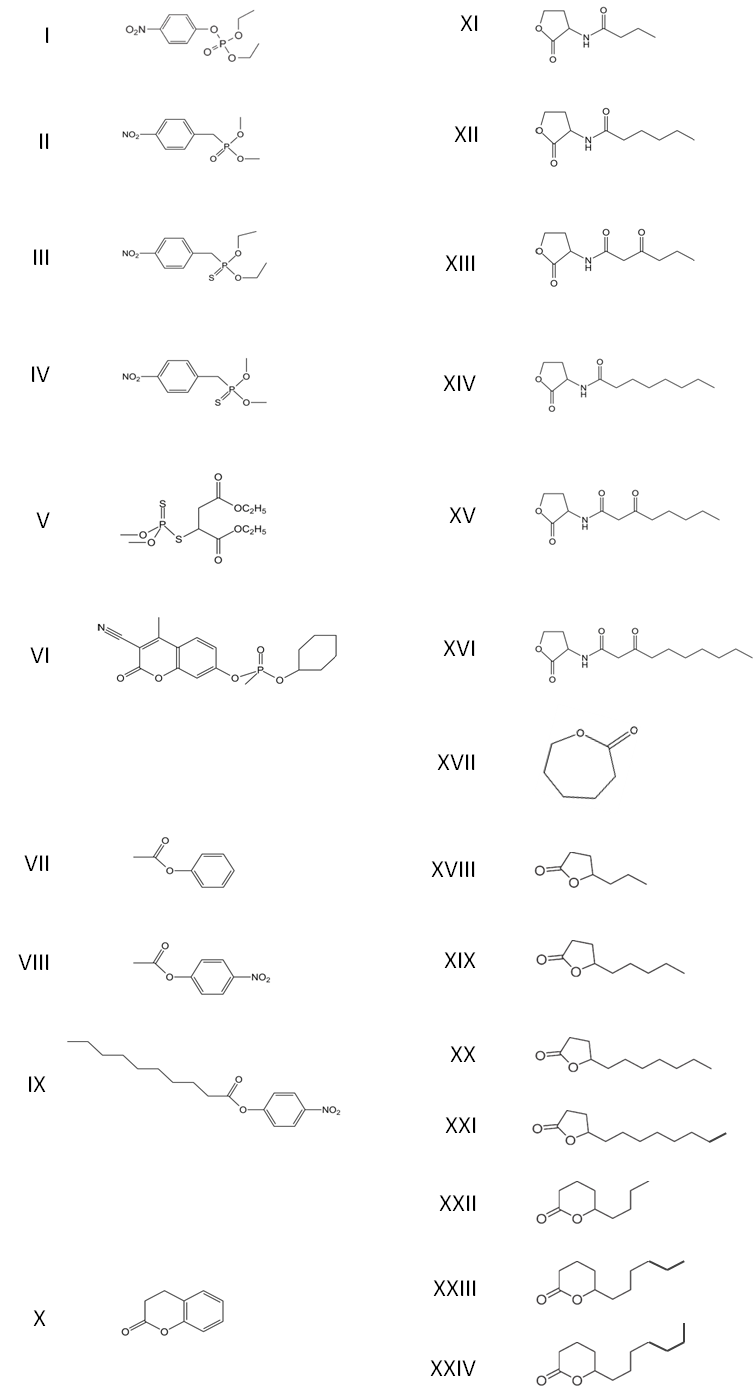
**

**Figure S1:** Chemical structure of phosphoesters (I-VI), esters (VII-IX) and lactones (X-XXIV)

Chemical structure of ethyl-paraoxon (I), methyl-paraoxon (II), ethyl-parathion (III), methyl-parathion (IV), malathion (V), CMP-Coumarin (VI), phenyl-acetate (VII), *p*NP-acetate (VIII), *p*NP-decanoate (IX), dihydrocoumarin (X), C4-AHL (XI), C6-AHL (XII), 3-oxo-C6-AHL (XIII), C8-AHL (XIV), 3-oxo-C8-AHL (XV), 3-oxo-C10-AHL (XVI), ε-caprolactone (XVII), γ-heptanolide (XVIII), Nonanoic-γ-lactone (XIX), Undecanoic-γ-lactone (XX), Dodenanoic-γ-lactone (XXI) Nonanoic-δ-lactone (XXII), Undecanoic-δ-lactone (XXIII) and Dodenanoic-δ-lactone(XXIV)

**
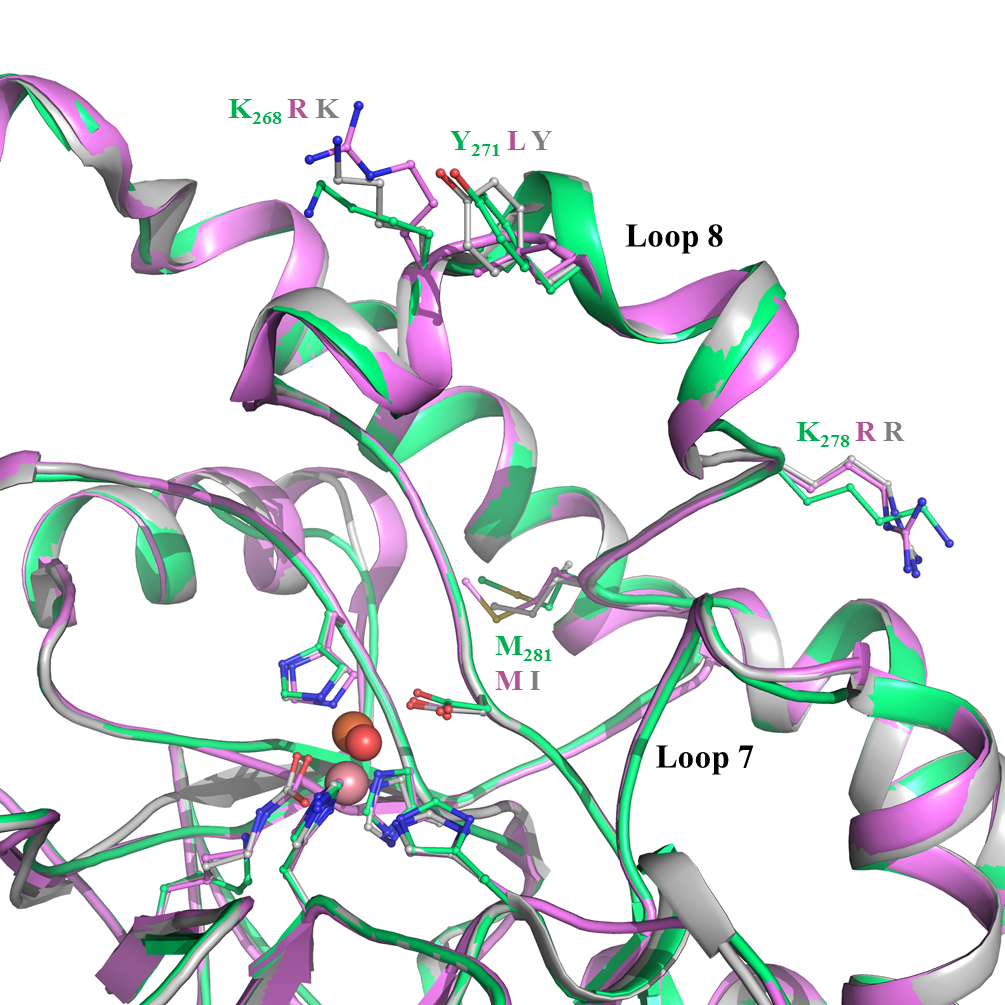
**

**Figure S2:** Superposition of *Sso*Pox, *Sis*Lac and *Sac*Pox structural models.

The substitutions in loop 8 that are not directly involved in the active site are highlighted. Active site view of superimposed *Sso*Pox structure (grey), *Sis*Lac structure (purple) and the *Sac*Pox model (green). Several loop 8 residues are represented as sticks. Numbering is made according to *Sac*Pox sequence.

**Table S1:** Accession numbers of the sequences used in the phylogeny study

| **Sequence** | **NCBI Accession number** |
| --- | --- |
| ***Gs*P** (*Geobacillus thermodenitrificans*) | [YP_001125472.1](http://www.ncbi.nlm.nih.gov.gate1.inist.fr/protein/138895019?report=genbank&log$=prottop&blast_rank=1&RID=CRTY14SU015) |
| ***Gk*L** (*Geobacillus kaustophilus*) | [YP_147359.1](http://www.ncbi.nlm.nih.gov.gate1.inist.fr/protein/56420041?report=genbank&log$=prottop&blast_rank=1&RID=CRHWGZ82014) |
| ***Dr*OPH** (*Deinococcus radiodurans*) | [NP_294654.1](http://www.ncbi.nlm.nih.gov.gate1.inist.fr/protein/15805954?report=genbank&log$=prottop&blast_rank=1&RID=CRJWPA64014) |
| **PTE** (*Brevundimonas diminuta*) | GI:13786715 |
| **PTEflavob** (*Flavobacterium* sp.) | [AAV39527.1](http://www.ncbi.nlm.nih.gov.gate1.inist.fr/protein/54778533?report=genbank&log$=prottop&blast_rank=1&RID=CRWJH9VM014) |
| **PTEAgrobac** (*Agrobacterium tumefaciens*) | [AAK85308.1](http://www.ncbi.nlm.nih.gov.gate1.inist.fr/protein/15212234?report=genbank&log$=prottop&blast_rank=1&RID=CRWJH9VM014) |
| **opdA** (*Agrobacterium radiobacter*) | GI:167744959 |
| **PLLBreviba** (*Brevibacterium mcbrellneri*) | [WP_005881372.1](http://www.ncbi.nlm.nih.gov.gate1.inist.fr/protein/492547542?report=genbank&log$=prottop&blast_rank=1&RID=CRWJH9VM014) |
| **PLLDermaco** (*Dermacoccus* sp.) | [WP_006945246.1](http://www.ncbi.nlm.nih.gov.gate1.inist.fr/protein/494002697?report=genbank&log$=prottop&blast_rank=1&RID=CRWJH9VM014) |
| **AhlA**(*Rhodococcus erythropolis*) | [WP_003943005.1](http://www.ncbi.nlm.nih.gov.gate1.inist.fr/protein/490040601?report=genbank&log$=prottop&blast_rank=1&RID=CRV1400P014) |
| **QsdA** (*Rhodococcus erythropolis*) | [ABQ42704.1](http://www.ncbi.nlm.nih.gov.gate1.inist.fr/protein/146742384?report=genbank&log$=prottop&blast_rank=2&RID=CRV1400P014) |
| **PLLRhodoco** (*Rhodococcus jostii*) | [YP_701486.1](http://www.ncbi.nlm.nih.gov.gate1.inist.fr/protein/111018514?report=genbank&log$=prottop&blast_rank=1&RID=CRWJH9VM014) |
| **PLLStrepto** (*Streptosprangium roseum*) | [YP_003338238.1](http://www.ncbi.nlm.nih.gov.gate1.inist.fr/protein/271964042?report=genbank&log$=prottop&blast_rank=1&RID=CRWJH9VM014) |
| **MCP** (*Mycobacterium avium* subsp. *paratuberculosis* K-10) | [NP_962602.1](http://www.ncbi.nlm.nih.gov.gate1.inist.fr/protein/41409766?report=genbank&log$=prottop&blast_rank=1&RID=CRUJVE7T014) |
| **PPH** (*Mycobacterium tuberculosis*) | [NP_214744.1](http://www.ncbi.nlm.nih.gov.gate1.inist.fr/protein/15607371?report=genbank&log$=prottop&blast_rank=1&RID=CRUB49SG014) |
| **PLLMycobCD** (*Mycobacterium tuberculosis*) | [NP_214744.1](http://www.ncbi.nlm.nih.gov.gate1.inist.fr/protein/15607371?report=genbank&log$=prottop&blast_rank=1&RID=CRWJH9VM014) |
| **PLLMycbovi** (*Mycobacterium bovis*) | [NP_853900.1](http://www.ncbi.nlm.nih.gov.gate1.inist.fr/protein/31791407?report=genbank&log$=prottop&blast_rank=1&RID=CRWJH9VM014) |
| ***SacPox*** *(Sulfolobus acidocaldarius*) | [YP_256726.1](http://www.ncbi.nlm.nih.gov.gate1.inist.fr/protein/70607856?report=genbank&log$=prottop&blast_rank=1&RID=CRK526EE014) |
| ***Sis*Lac** (*Sulfolobus islandicus*) | [YP_002828495.1](http://www.ncbi.nlm.nih.gov.gate1.inist.fr/protein/227826716?report=genbank&log$=prottop&blast_rank=1&RID=CRK7WZT3015) |
| ***Sso*Pox** (*Sulfolobus solfataricus*) | [NP_343863.1](http://www.ncbi.nlm.nih.gov.gate1.inist.fr/protein/15899258?report=genbank&log$=prottop&blast_rank=1&RID=CRK238W5015) |
| **Symbact** (*Symbiobacterium thermophilum*) | [YP_074383.1](http://www.ncbi.nlm.nih.gov.gate1.inist.fr/protein/51891692?report=genbank&log$=prottop&blast_rank=1&RID=CRWJH9VM014) |
| **PHP_E** (*Escherichia coli*) | YP_001723339.1 |
| **PHP_P** (*Photorhabdus asymbitoca*) | [YP_003041416.1](http://www.ncbi.nlm.nih.gov.gate1.inist.fr/protein/253990060?report=genbank&log$=prottop&blast_rank=1&RID=CRWJH9VM014) |
| **PHP_Y** (*Yersinia aldovae*) | [WP_004702383.1](http://www.ncbi.nlm.nih.gov.gate1.inist.fr/protein/490840308?report=genbank&log$=prottop&blast_rank=1&RID=CRWJH9VM014) |
| **PHP_X** (*Xenorhabdus bovienii*) | [YP_003466084.1](http://www.ncbi.nlm.nih.gov.gate1.inist.fr/protein/290473219?report=genbank&log$=prottop&blast_rank=1&RID=CRWJH9VM014) |
| **RTX_K1** (*Klebsellia pneumoniae*) | [YP_001335395.1](http://www.ncbi.nlm.nih.gov.gate1.inist.fr/protein/152970286?report=genbank&log$=prottop&blast_rank=1&RID=CRWJH9VM014) |
| **RTX_K2** (*Klebsellia variicola*) | EGH93047.1 |
| **RTX_P** (*Pseudomonas syringiae*) | [WP_005782653.1](http://www.ncbi.nlm.nih.gov.gate1.inist.fr/protein/492229445?report=genbank&log$=prottop&blast_rank=1&RID=CRWJH9VM014) |
| **RTX_3K** (*Rhodobacter sphaeroides*) | [YP_001045290.1](http://www.ncbi.nlm.nih.gov.gate1.inist.fr/protein/126464177?report=genbank&log$=prottop&blast_rank=1&RID=CRWJH9VM014) |

**Table S2**: Sequence identity matrix

|  | ***Bd*PTE** | ***Sso*Pox** | ***Sis*Lac** | ***Sac*Pox** | **PPH** | **MCP** | **AhlA**  **QsdA** | ***Dr*OPH** | ***Gk*L** | ***Gs*P** |
| --- | --- | --- | --- | --- | --- | --- | --- | --- | --- | --- |
| ***Bd*PTE** | **-** | **32.8** | **32.2** | **33.8** | **34.8** | **35.7** | **28.9** | **30.34** | **27.2** | **27.2** |
| ***Sso*Pox** |  | - | 91.4 | 76.1 | 39.2 | 37.6 | 37.9 | 28.34 | 33.8 | 32.9 |
| ***Sis*Lac** |  |  | - | 76.1 | 37.6 | 36.31 | 36.6 | 27.4 | 33.8 | 32.2 |
| ***Sac*Pox** |  |  |  | - | 39.8 | 39.2 | 38.5 | 30.6 | 33.1 | 32.2 |
| **PPH** |  |  |  |  | - | 92 | 59 | 31.6 | 32.7 | 32.4 |
| **MCP** |  |  |  |  |  | - | 58.7 | 31.6 | 32.7 | 32.7 |
| **AhlA/QsdA** |  |  |  |  |  |  | - | 32 | 30.8 | 31.1 |
| ***Dr*OPH** |  |  |  |  |  |  |  | - | 58.8 | 60.7 |
| ***Gk*L** |  |  |  |  |  |  |  |  | - | 90.4 |
